# The interphase period “germination–heading” of 8x and 6x triticale with different dominant Vrn genes

**DOI:** 10.18699/VJ21.071

**Published:** 2021-10

**Authors:** P.I. Stepochkin, A.I. Stasyuk

**Affiliations:** Siberian Research Institute of Plant Production and Breeding – Branch of the Institute of Cytology and Genetics of the Siberian Branch of the Russian Academy of Sciences, Krasnoobsk, Novosibirsk region, Russia; Institute of Cytology and Genetics of the Siberian Branch of the Russian Academy of Sciences, Novosibirsk, Russia

**Keywords:** octaploid, hexaploid triticale, interphase period “germinarion–heading”, Vrn-1 genes, hybrids, октаплоидные, гексаплоидные тритикале, межфазный период «всходы–колошение»;, гены Vrn-1, гибриды

## Abstract

The existing spring forms of wheat-rye amphiploids are characterized by late maturity due to the long duration of the interphase period “germination–heading”. The manifestation of this trait is inf luenced by Vrn-1 genes. Their dominant alleles also determine the spring type of development. The results of studying the interphase period “germination– heading” of spring octaploid and hexaploid forms of triticale created for use in research and breeding programs under the conditions of forest-steppe of Western Siberia are given in this article. The interphase period of the primary forms 8xVrnA1, 8xVrnB1 and 8xVrnD1 obtained by artif icial doubling of the chromosome number of the wheat-rye hybrids made by pollination of three lines of the soft wheat ‘Triple Dirk’ – donors of different dominant Vrn-1 genes – by a winter rye variety ‘Korotkostebel’naya 69’ was determined under the f ield conditions in the nursery of octaploid (8x) triticale. In the nursery of hexaploid triticale, this trait was studied in the populations of hybrids obtained by hybridization of these three primary forms of octaploid triticale with the hexaploid winter triticale variety ‘Sears 57’. In the offspring of crossing 8хVrnD1 × ‘Sears 57’, spring genotypes of 6x triticale bearing Vrn-D1 were selected. This fact was determined by PСR. It means that the genetic material from the chromosome of the f ifth homeologous group of the D genome of the bread wheat is included in the plant genotypes. This genome is absent in the winter 6x triticale ‘Sears 57’. The grain content of spikes of the created hexaploid forms of triticale is superiour to that of the maternal octaploid triticale forms. It was shown that plants of the hybrid populations 8xVrnA1 × ‘Sears 57’ and 8xVrnD1 × ‘Sears 57’ carrying the dominant alleles Vrn-A1a and Vrn-D1a, respectively, have a shorter duration of the “germination–heading” interphase period than the initial parental forms of primary 8x triticale. The short interphase period of “germination–heading” of the 6x triticale is a valuable breading trait for the creation of early maturing and productive genotypes of triticale.

## Abstract (ru)

Существующие коллекционные формы яровых пшенично-ржаных амфиплоидов характеризуются позднеспелостью из-за большой продолжительности межфазного периода «всходы–колошение». На проявле- ние этого признака влияют гены Vrn-1, аллели которых в доминантном состоянии обусловливают яровой тип развития. В статье приведены результаты изучения межфазного периода «всходы–колошение» у яровых окта- и гексаплоидных форм тритикале, создаваемых для использования в исследовательских и селекционных програм- мах в условиях лесостепи Западной Сибири. Исследования проводили в питомнике октаплоидных (8х) тритикале в полевых условиях у первичных форм 8хVrnA1, 8хVrnD1 и 8хVrnВ1, полученных искусственным удвоением чис- ла хромосом пшенично-ржаных гибридов от опыления пыльцой озимой ржи (сорт Короткостебельная 69) трех линий мягкой пшеницы Triple Dirk – доноров разных доминантных генов Vrn-1. В питомнике гексаплоидных (6х) тритикале изучали этот признак растений в популяциях гибридов от скрещиваний трех форм первичных окта- плоидных тритикале с гексаплоидным озимым сортом тритикале Сирс 57. С помощью молекулярных маркеров у гибридов определен аллельный состав генов Vrn-1. В потомстве, полученном от скрещивания 8хVrnD1 × Сирс 57, выделены и определены методом ПЦР генотипы яровых растений 6х тритикале с доминантным геном Vrn-D1. Данный факт свидетельствует о включении в них генетического материала хромосомы пятой гомеологичной группы генома D мягкой пшеницы, входящего в геномный состав октаплоидного тритикале. Этот геном отсут- ствует в озимом 6x тритикале Сирс 57. У созданных гексаплоидных форм тритикале озерненность колоса была лучше, чем у материнских октаплоидных. Показано, что растения из гибридных популяций 8хVrnА1 × Сирс 57 и 8хVrnD1 × Сирс 57, несущие доминантные аллели Vrn-А1a и Vrn- D1a соответственно, обладают более короткой продолжительностью межфазного периода «всходы–колошение», чем исходные родительские формы первич- ных 8х тритикале. Короткий межфазный период «всходы–колошение» у полученных 6х тритикале является се- лекционно ценным признаком для создания раннеспелых и продуктивных генотипов тритикале.

## Introduction

Hundreds of winter and spring varieties and collection forms of triticale (×Triticosecale Wittmack) or wheat-rye amphiploid (WRA) with genomes of wheat (Triticum spp.) and rye (Secale spp.) have been made for more than 130-year history of this artificial crop. According to the latest data of the world organization FAO, in 2017, the total area of this crop reached almost 4.17 million hectares and grain production was 15.6 million tons. In the Russian Federation the area of crops decreased up to 171.7 thousand hectares in 2017, compared with the maximum value 274.5 thousand hectares in 2014. Grain yield for those years amounted to 500.7 thousand tons and 654.1 thousand tons, respectively (http://www.fao.org/faostat/ru/#data/QC/visualize) This circumstance is due to a decrease in breeding work and creation of varieties of wheatrye amphiploids in the Russian Federation in recent years.

Hexaploid (6x) forms of triticale (BuBuAARR, 2n = 42) are mainly used for agricultural practice. They are more cytogenetically stable and fertile compared to octaploid (8x) (BuBuAADDRR, 2n = 56) ones (Lukaszewski, Gustafson, 1987). However, there are some reports of successful cultivation of 8x triticale (Cheng, Murata, 2002).

In Siberia, this crop has not yet been cultivated on a large scale, since selection of spring triticale forms has not been conducted. Spring triticale samples from the world collection of VIR are late-maturing and winter varieties of European selection do not have good winter hardiness and often do not give good grain yield under severe climatic conditions of Siberia. Two winter short-stemmed varieties Sears 57 and Cecad 90 have been created in Siberian Research Institute of Plant Production and Breeding – Branch of the Federal Research Center the Institute of Cytology and Genetics of the Siberian Branch of the Russian Academy of Sciences (SibRIPP&B – Branch of ICG SB RAS) for grainforage use. They occupy only several thousands hectares. More than a dozen of spring varieties have been created in Russia (Tyslenko et al., 2016), but there are no Siberian varieties of spring triticale yet, although spring crops yield yearly, unlike winter ones. In order to carry out breeding work with spring triticale successfully, it is necessary to comprehensively study the characteristics associated with the productivity and adaptability of plants, including those related to the type and duration of plant development.

The type of plant development (spring, winter, alternative), and the duration of the growing period are controlled by Vrn (response to vernalization) genes. The key role in wheat species is played by dominant Vrn-1 genes: Vrn-A1, Vrn-B1, and Vrn-D1 (Yan et al., 2003; Muterko et al., 2015,

2016; Shcherban et al., 2015; Dixon et al., 2019). They are in the long arms of 5A, 5B, and 5D chromosomes of three genomes of soft wheat A, B, and D, respectively. There are also the Vrn- D4 gene located in the centromere region of 5D chromosome (Yoshida et al., 2010; Kippes et al., 2015) and the Vrn-B3 gene located in the short arm of chromosome 7B (Yan et al., 2006). Studies have revealed the presence of several alleles in each of the Vrn-1 genes (Yan et al., 2004; Fu et al., 2005; Shcherban et al., 2012; Muterko et al., 2015). The dominant state of any of these genes leads to the spring type of development, and the recessive state leads to the winter type (Pugsley, 1971; Worland, 1996; Yan et al., 2003, 2004, 2006; Fu et al., 2005). The type of plant development of rye is controlled by the Vrn-R1 gene located in the long arm of the chromosome 5R (Plaschke et al., 1993).

Vrn-1 genes of spring wheat varieties mainly determine the duration of the phases from tillering to tube formation. Duration of the period from germination to heading depends on the allelic state of Vrn-1 genes. It was shown that the plants containing Vrn-B1c allele formed spikes earlier than those with Vrn-B1a allele (Emtseva et al., 2013). The expression of Vrn-A1a allele leads to earlier earing than that of Vrn-B1a or Vrn-B1c allele (Kruchinina et al., 2017). The dominant gene Vrn-A1 of soft wheat T. aestivum L. has the greatest effect and the dominant gene Vrn-B1 has the smallest one (Košner, Pánková, 2004). In octaploid triticale lines created on the basis of almost isogenic on dominant genes Vrn-1 lines of soft wheat Triple Dirk, the plants with Vrn-A1a and Vrn-D1a genes formed spikes earlier than those with the Vrn-B1a gene (Stepochkin, Emtseva, 2017).

The purpose of this article is to study the duration of the interphase period “germination–heading” of created in SibRIPP&B – Branch of ICG SB RAS spring octaploid and hexaploid triticale forms having different dominant Vrn-1 genes under conditions of the forest-steppe of Western Siberia.

## Materials and methods

The duration of the interphase period “germination–heading” of octaploid (8x) and hexaploid (6x) triticales with different dominant Vrn-1 genes affecting the duration of the vegetation period of plants was studied in generations: F1 – in 2014, F3 – in 2016, F4 – in 2017, F5 – in 2018 and F6 – in 2019.

Three primary 8x triticale forms were made in SibRIPP&B – Branch of ICG SB RAS by crossing almost isogenic lines of soft wheat Triple Dirk D, Triple Dirk B and Triple Dirk E (Pugsley, 1971, 1972) with winter diploid rye variety Korotkostebel’naya 69 and by subsequently doubling the some number in wheat-rye hybrids (Stepochkin, 2009, 2017). The wheat lines are the sources and donors of dominant genes Vrn-A1, Vrn-B1 and Vrn-D1, respectively. The allelic compositions of Vrn-1 genes of three 8x WRA are Vrn-A1a, vrn-B1, vrn-D1, vrn-R1 (8xVrnA1); vrn-A1, Vrn-B1a, vrn-D1, vrn-R1 (8xVrnB1); vrn-A1, vrn-B1, Vrn-D1a, vrn-R1 (8xVrnD1).

**Fig. 1.**
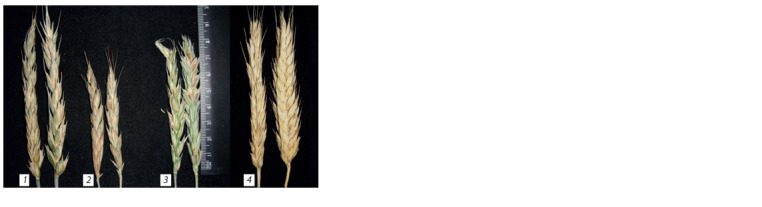
Spikes of spring octaploid triticale plants: 8хVrnA1 (1), 8хVrnD1 (2),8хVrnB1 (3) and winter hexaploid triticale Sears 57 (4).

Spring hexaploid forms of triticale were made by selecting early-maturing plants in the offspring of F3–F4 hybrids between primary 8x WRA and winter 6x triticale Sears 57 carrying recessive genes vrn-A1, vrn-B1, vrn-R1 (Fig. 1). The allelic composition of Vrn-1 genes in plants of hybrid populations and parent forms was determined by PCR using allele-specific primers. The structure of primers to Vrn-1 genes and the conditions of PCR are described in articles (Potokina et al., 2012; Likhenko et al., 2015).

Genomic DNA was isolated according to the previously described method (Likhenko et al., 2015). PCR was performed on a BIO-RAD T-100 Thermal Cycler (USA) amplificator in a total reaction mixture of 20 μl, including DNA (50–100 ng/ μl) – 1 μl, 10× buffer for Taq polymerase (650 mM Tris-HCl (pH 8.9); 160 mM (NH4)2SO4; 25 mM MgCl2; 0.5 % Tween 20) – 2 μl, dNTPs – 2 μl, direct and reverse primer – 0.5 μl each, Taq polymerase (1 unit/μl) – 1 μl, H2O – up to the final volume of 20 μl. The separation of PCR products was performed by electrophoresis in 1 % agarose gel with the addition of ethidium bromide.

Sowing in the field soil was carried out by hand in the third decade of May (May 21–24 in different years, depending on the weather) in rows of 0.8 m long, 50 seeds in a row on the isolated from other grain crops experimental plot of SibRIPP&B – Branch of ICG SB RAS, where a three–field crop rotation was maintained: vegetables – fallow soil – triticale. During the growing season, phenological observations and evaluations were carried out. Statistical processing of the results was performed using the Student’s t-test (Dospekhov, 1985).

## Results

The evaluation of plants in populations of primary octaploid WRA showed that the duration of the interphase period “germination– heading” in triticale lines 8xVrnA1 and 8xVrnD1 in 2018 and 2019 was shorter than that in 2014, 2016 and 2017 (Table 1). In 2019, triticale 8xVrnA1 had the shortest “germination– heading” period (52.9 days) among all octaploid WRA, while 8xVrnB1 had the longest one (72.5 days). This period of the maternal line 8xVrnD1 lasted 53.8 days. In 2019 hexaploid plants of the hybrid population 8xVrnA1 × 6x Sears 57 in comparison with other hexaploid forms had the shortest duration of this period (47.3 days) and plants of the population 8xVrnB1 × 6x Sears 57 had the longest one (57.8 days). Unlike the maternal forms, this period at 6x level was 6 and 14 days shorter. 6x plants made by crossing 8xVrnD1 × Sears 57 had the period of development before earing of 53.4 days and did not significantly differ from the spring octaploid parent. When comparing the data of all years of research, one can note that the selection of the most early-maturing plants in each generation led to a significant reduction of the duration of the period from germination to heading of both hexaploid and maternal octaploid triticale forms except for the parental form 8xVrnB1. It did not show significant changes in the duration of this period during all years of research.

**Table 1.**
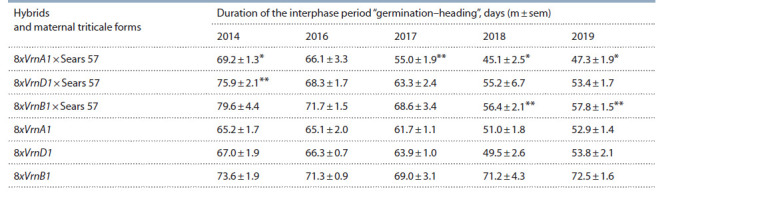
Duration of the interphase period “germination–heading” of hybrid hexaploid and maternal octaploid triticale plants with different dominant Vrn-1 genes * p <0.05; ** p <0.01 – signif icant differences between a hybrid and its parental form of 8x triticale.

**Fig. 2.**
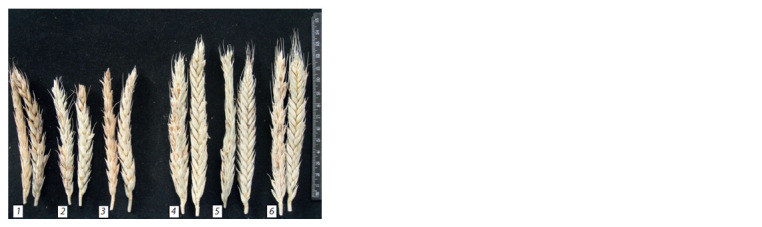
Ears of triticale plants: octaploid 8хVrnA1 (1), 8хVrnB1 (2),8хVrnD1 (3); hexaploid made from crossing 8хVrnA1 × Sears 57 (4),8xVrnB1 × Sears 57 (5), 8xVrnD1 × Sears 57 (6).

Ear morphology of hexaploid triticale plants differs from that of the original octaploid forms (Fig. 2). All octaploid triticale lines are awnless, and the hexaploid forms have, like the paternal winter variety Sears 57, small rudiments of the awns, mainly at the end of the spike. The octaploid amphiploid 8xVrnD1 as well as the hexaploid 6xVrnD1 derived from it (hybrid 8xVrnD1 × Sears 57) have hairy spikes – a trait inherited from Triple Dirk E wheat (VrnD1).

For practical use, it is important to note that ears of all hexaploid plants are denser and contain more kernels than those of 8x triticale (Table 2). In 2019, the number of kernels in ears of 6x forms varied from 25.8 grains of hybrids 8xVrnB1 × Sears 57 up to 36.4 grains of hybrids 8xVrnA1 × Sears 57, and in octaploid lines – from 9.1 grains in ears of 8xVrnD1 to 16.4 grains in ears of 8xVrnA1. Weight of grains from ear varied in 6x forms from 0.76 ± 0.10 to 1.28 ± 0.21 g, and in 8x forms – from 0.24 ± 0.03 to 0.50 ± 0.13 g. In addition, grain unit in hexaploid forms is slightly higher than that in 8x WRA. No significant differences between hexaploid and octaploid triticale forms were found for the weight of 1000 grains.

**Table 2.**
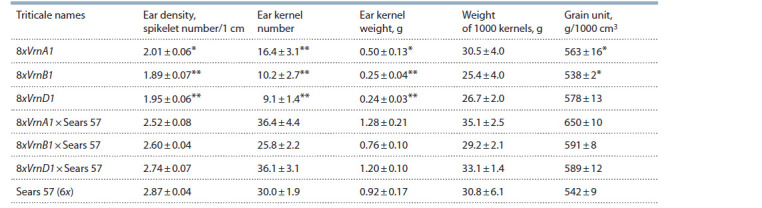
Some ear quantitative characteristics of 8x and 6x triticale with different Vrn-1 genes, 2019 * p <0.05; ** p < 0.01 – signif icant differences between a hybrid and its parental form of 8x triticale.

The combination of early ripeness, which is largely due to the duration of the interphase period “germination–heading”, and the number of grains of ear makes two hexaploid forms 8xVrnA1 × Sears 57 and 8xVrnD1 × Sears 57 promising for further breeding work.

**Fig. 3.**
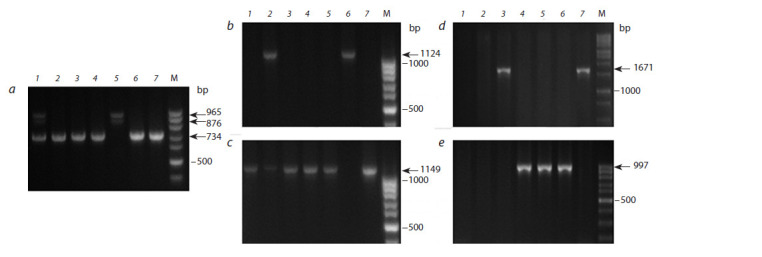
Amplif ication of the PCR products using primers for Vrn-1 genes in hexaploid hybrids of triticale and parental forms: (a) Vrn-A1a (965 + 876 bp) and vrn-A1 (734 bp); (b) Vrn-B1a (1124 bp); (c) vrn-B1 (1149 bp); (d) Vrn-D1a (1671 bp); (e) vrn-D1 (997 bp). 1–3 – hybrids: 8хVrnА1 × Sears 57, 8хVrnВ1 × Sears 57 and 8хVrnD1 × Sears 57, respectively; 4 – winter variety Sears 57; 5–7 – spring octaploid forms: 8хVrnA1, 8хVrnB1 and 8хVrnD1, respectively; M – marker of the length of DNA fragments (a–c, e – 100 bp ladder; d – 1000 bp ladder).

The selected early-maturing hexaploid plants were analyzed by PCR using allele-specific primers for Vrn-1 genes. Their parents – the winter variety triticale Sears 57 and the octaploid maternal forms were taken for comparison (Fig. 3). The analysis showed that the winter variety Sears 57 carries recessive alleles vrn-A1, vrn-B1 and vrn-D1. The maternal forms had the following allelic composition: 8xVrnA1 – Vrn- A1a, vrn-B1, vrn-D1; 8xVrnB1 – vrn-A1, Vrn-B1a, vrn-D1; 8xVrnD1 – vrn-A1, vrn-B1, Vrn-D1a. The offspring from the 8xVrnA1 × Sears 57 cross was heterozygous on the Vrn-A1 gene because they contained two alleles – Vrn-A1a and vrn-A1. In addition, a recessive allele vrn-B1 was revealed, and the alleles of the Vrn-D1 gene were not determined due to the lack of an amplification product. Plants of the hybrid population 8xVrnB1 × Sears 57 have a recessive allele vrn-A1, and the Vrn-D1 gene was not amplified in them. As for the Vrn-B1 gene, the plants are heterozygous and have two alleles Vrn-B1a and vrn-B1. Two recessive alleles – vrn-A1, vrn-B1, and one dominant allele – Vrn-D1a – were identified in plants obtained from crossing 8xVrnD1 × Sears 57.

## Discussion

Secondary spring 6x triticale breeding samples possess dominant alleles of Vrn-1 genes and were made by hybridization of primary 8x WRA carrying dominant alleles of Vrn-1 genes with a winter 6x WRA carrying recessive alleles. At the 6x level in the offspring of hybrids in all the studied generations, their Vrn-1 genes retain almost the same ranking (8xVrnA1 × 6x Sears 57 > 8xVrnD1 × 6x Sears 57 > 8xVrnB1 × 6x Sears 57) as at the 8x level in triticale (8xVrnA1 ≥ 8xVrnD1 > 8xVrnB1) in terms of the effect on the reduction of the “germination–heading” period. The original Triple Dirk lines have the same ranking of these genes (Stepochkin, 2009; Stepochkin, Emtseva, 2017). Thus, the effect of the dominant alleles Vrn-A1a and Vrn-D1a leads to a shorter interphase period compared to the effect of the Vrn-B1a allele. It is known that in addition to the Vrn-D1 gene, the Vrn-D4 gene localized in the chromosome 5D can significantly affect the duration of the period from germination to earing (Kippes et al., 2014). Theoretically, it is possible that along with the Vrn-D1 gene, a Vrn-D4 gene can be inserted. However, we exclude this possibility, because to make primary octaploid triticales, we used the Vrn-isogenic wheat lines Triple Dirk D, Triple Dirk B, and Triple Dirk E, carrying, respectively, only the Vrn-A1, Vrn-B1, and Vrn-D1 genes. A comparison of a set of 8x triticale lines and 6x samples from the VIR world collection showed that the interphase period “germination–heading” of hexaploid triticales is shorter (Stepochkin, Emtseva, 2017). There is an assumption that reducing the level of ploidy can reduce the duration of the period “from germination to heading” in wheatrye amphiploids. In particular, it was reported that within the crossing combination, octaploid lines formed ears later than hexaploid ones (Kaminskaya et al., 2005).

The hexaploid paternal variety Sears 57 (genomic formula BuBuAARR), has a winter type of development. All its vrn-1 genes have recessive alleles. The maternal forms are three spring octaploid triticale lines (genomic formula BuBuAADDRR). Each of them carries one dominant gene: 8xVrnA1 carries a VrnA1a allele on the chromosome 5A, 8xVrnB1 contains a VrnB1a allele on the chromosome 5B, 8xVrnD1 has a Vrn-D1a allele on the 5th chromosome of D genome. It was assumed that in 8xVrnD1 × Sears 57 hybrids in subsequent generations, starting from F2, the chromosomes of the haploid D genome would be lost during the process of meiosis, and the share of winter plants in the hybrid populations would increase. As a result, in the older generations there would be only winter hexaploid forms with the chromosome number 42 without the haploid genome D and without the dominant allele Vrn-D1a. The facts of complete elimination of chromosomes of D genome in such types of crossing are known (Hao et al., 2013). However, by selecting spring plants we were able to create up to the fourth generation populations of 6x forms that could begin transition to the generative development after spring sowing without vernalization. Molecular genetic analysis using the PCR method showed the presence of the dominant Vrn-D1a allele in these forms (see Fig. 3). This means that either as a result of chromosome substitution or translocation, the Vrn-D1 gene remained in the complex genome of hexaploid plants. Some researchers report inclusion of a genetic material of the wheat genome D in the genome of hexaploid triticale forms (Kaminskaya et al., 2005). Unlike plants of 8xVrnD1 × Sears 57 population, hexaploid triticale forms made by crosses 8xVrnA1 × Sears 57 and 8xVrnB1 × Sears 57 do not contain any Vrn-D1 allele, as it was shown by molecular analysis with primers to Vrn-D1 gene, although the maternal lines contain a recessive vrn-D1 allele. The lack of amplification is probably due to the elimination of chromosomes of the D genome.

It is known that octaploid triticales are cytogenetically unstable. As a result of disturbances in meiosis, gametes with an unbalanced number of chromosomes are formed, which leads to appearance of aneuploid plants in 8x WRA populations (Vettel, 1960a, b; Krolow, 1962, 1963). Hexaploid triticale plants with dominant Vrn-1 genes may arise as a result of spontaneous depoliploidization of octaploid WRA carrying these genes. This process is accompanied by the predominant elimination of the chromosomes of D genome of soft wheat in octaploid WRA. At the end of this process, stable 6x triticales appear, which was found in populations of a number of 8x triticales (Stepochkin, 1978; Li et al., 2015).

## Conclusion

The presented results showed that the populations of spring octaploid triticales made and maintained at SibRIPP&B – Branch of ICG SB RAS are donors of different dominant Vrn-1 genes. These populations are used to produce new forms of 8x and 6x WRA and for breeding process. In the hexaploid triticale forms made on their basis, the allelic composition of the Vrn-1 genes was determined using molecular genetic analysis. It was found that plants from the populations of 8xVrnA1 × Sears 57 and 8xVrnB1 × Sears 57 have genes Vrn- A1 and Vrn-B1 in a heterozygous state, so it is necessary to conduct further selection to make homozygous genotypes. In the created hexaploid forms of triticale, the grain number from ear is higher than that in the original octaploid lines. It is shown that the plants from the hybrid populations 8xVrnA1 × Sears 57 and 8xVrnD1 × Sears 57, carrying the dominant alleles Vrn-A1a and Vrn-D1a, respectively, have a shorter duration of the interphase period “germination–heading” than the original parent forms of the primary 8x triticale, which is a breeding-valuable feature for the creation of early-maturing and productive genotypes of triticale.

## Conflict of interest

The authors declare no conflict of interest.
